# Symptomatic pelvic hematoma following hysterectomy: risk factors, bacterial pathogens and clinical outcome

**DOI:** 10.1186/s12905-020-01140-0

**Published:** 2020-12-09

**Authors:** Henry H. Chill, Itshak Amsalem, Gilad Karavani, Sharon Amit, Abraham Benshushan, David Shveiky

**Affiliations:** 1 Section of Female Pelvic Medicine and Reconstructive Surgery, Department of Obstetrics and Gynecology, Faculty of Medicine, Hadassah Medical Center, Hebrew University of Jerusalem, PO Box 12000, Ein Kerem, Jerusalem, Israel; 2 Department of Obstetrics and Gynecology, Hadassah – Hebrew University Medical Center, Ein Kerem, Jerusalem, Israel; 3 Hebrew University Medical School, Jerusalem, Israel; 4 Clinical Microbiology, The Chaim Sheba Medical Center, Affiliated to the Tel-Aviv University Sackler School of Medicine, Ramat-Gan, Israel

**Keywords:** Hysterectomy, Infected hematoma, Pelvic hematoma, Pelvic organ prolapse, Antibiotic prophylaxis

## Abstract

**Background:**

Pelvic hematoma is a common finding following hysterectomy which at times may become infected causing substantial morbidity. The aim of this study was to describe the incidence, clinical manifestation and identify risk factors for infected pelvic hematoma. We also attempted to identify specific bacterial pathogens which may cause this phenomenon.

**Methods:**

We conducted a retrospective cohort study at a tertiary university teaching hospital. Included were all women who underwent hysterectomy and were diagnosed with a pelvic hematoma following surgery from 2013 to 2018. In an attempt to assess possible risk factors for infected pelvic hematoma women with asymptomatic pelvic hematoma were compared to women with an infected pelvic hematoma.

**Results:**

During the study period 648 women underwent hysterectomy at our medical center. Pelvic hematoma was diagnosed by imaging in 50 women (7.7%) including 41 women who underwent vaginal hysterectomy and 9 women who underwent abdominal hysterectomy. In 14 (2.2%) cases the hematoma became infected resulting in need for readmission and further treatment. Women who underwent vaginal surgery were more likely to return with infected pelvic hematoma compared to women who underwent open abdominal or laparoscopic surgery (4.5% vs. 1.1%, *p* < 0.05). In 8 women bacterial growth from hematoma culture was noted. *Enterococcus faecalis*, was the most abundant pathogen to be isolated in this sub-group.

**Conclusion:**

Vaginal route of hysterectomy is a risk factor for infected pelvic hematoma following hysterectomy. Most of these infections were caused by anaerobic bacteria which may not be sufficiently covered by current antibiotic prophylactic regimens.

## Background

Hysterectomy is nowadays one of the most common surgeries performed in the United States with an estimated one in nine women having to undergo the procedure during their lifetime [1]. Hysterectomy may be performed via abdominal or vaginal route with the former including open, laparoscopic and robotic surgery.

Pelvic hematoma is a common postoperative finding following hysterectomy, with an incidence ranging between 25 and 98% [2, 3]. While most of these hematomas are asymptomatic and may be discovered as an incidental finding during ultrasound exam, occasionally they may become infected causing pain, fever, foul smelling discharge and need for readmission. Previous studies have estimated the incidence of infected hematoma to be 6–9% most of which require intervention such as antibiotic treatment, surgical drainage and pain relief [2, 4].

Little is known regarding risk factors for development of infected hematoma after hysterectomy. Segev et al. reported on 28 women with symptomatic pelvic hematoma following vaginal hysterectomy. Vaginal surgical route was shown to be a risk factor for formation of such hematomas but no other risk factors were identified [4]. These findings are in contrast to a previous Cochrane review which did not find a significant difference with regard to infection between vaginal and abdominal routes but there was no direct reference to infected hematomas [5]. According to another study hematoma size may play an important role showing that hematomas larger than 5 cm were associated with fever [6].

Management of infected hematoma encompasses antibiotic treatment with or without drainage. Drainage may occur spontaneously but at times entails surgical intervention. Removal of vaginal cuff stitches, ultrasound guided percutaneous and transvaginal drainage and CT percutaneous guided approach are all viable treatment options [7]. Rarely, surgical exploration is necessary particularly when access to the infected area is limited or following failure of more conservative treatments.

Prevention of postoperative infection is comprised of proper cleansing of the surgical field, sterile surgical measures and antibiotic prophylaxis. Recommended prophylactic antibiotic treatment prior to hysterectomy includes a first generation cephalosporin [8]. Previous studies have failed to identify specific bacteria species which may be instrumental in formation of infected pelvic hematoma. To date it is unclear whether prophylactic treatment administered supplies adequate coverage addressing possible pathogens which may be involved.

The aim of this study was to describe the incidence, clinical manifestation and management of infected vaginal hematoma following hysterectomy and to evaluate risk factors for this phenomenon. We also attempted to identify specific bacteria causing infected hematoma and assess the suitability of prophylactic antibiotic treatment.

## Methods

We conducted a retrospective cohort study at a tertiary university teaching hospital. Included were all women who underwent hysterectomy (abdominal, laparoscopic or vaginal route) and were diagnosed with a pelvic hematoma following surgery from 2013 to 2018. The study was approved by the Hadassah Medical Center, Hebrew University institutional ethical review board (0603-17-HMO). No consent for participation was required.

Pelvic hematomas were diagnosed either during post-void residual urine testing performed postoperatively (routinely performed in our medical center following pelvic organ prolapse repair) or following sonographic evaluation of symptoms associated with a diagnosis of infected pelvic hematoma.

Electronic medical records of women who underwent hysterectomy during the study period were reviewed for presence of a hematoma on ultrasound exam following surgery. Excluded were women for whom medical records were substantially incomplete. Information collected from medical records of women diagnosed with a pelvic hematoma included demographic, preoperative, intraoperative and postoperative data. For women who had hysterectomy for Pelvic Organ Prolapse (POP), Pelvic Organ Prolapse Quantification System (POP-Q) measurements were recorded. Women with a pelvic hematoma who presented with symptoms of fever, pelvic pain, foul smelling discharge or were in need of surgical drainage were considered to have an infected pelvic hematoma unless an alternative diagnosis could explain their clinical state. Pain was assessed using a scale of one to ten with ten representing the most severe pain experienced. Foul discharge was assessed by the physician performing physical exam in the clinic or emergency room. Women were considered febrile following two measurements of 38.0 °C half an hour apart.

Surgeries were performed using similar antiseptic measures and sterile environment. All surgeries were performed by a senior physician with subspecialty training in either oncologic gynecology or urogynecology with the assistance of a resident. Prior to abdominal surgery, scrubbing using chlorhexidine gluconate 4% with detergent followed by alcohol-chlorhexidine solution (0.5% chlorhexidine–gluconate in 70% isopropyl alcohol) was implemented. For vaginal hysterectomy cleansing with iodine based solution was standard for all procedures. Women received prophylactic antibiotic treatment 30 min prior to skin incision which included in most cases a first generation cephalosporin. In certain cases, due to allergies alternative regimens were used.

In an attempt to assess possible risk factors for infected pelvic hematoma women with asymptomatic pelvic hematoma were compared to women with an infected pelvic hematoma. Postoperative data for women with an infected pelvic hematoma comprised of clinical presentation, laboratory findings including results of bacterial cultures, sonographic findings, treatment modality and clinical course following treatment. Furthermore, risk of infected pelvic hematoma according to surgical route (abdominal vs. vaginal) was assessed.

Sub-group analysis focusing on women who underwent vaginal hysterectomy, all of which underwent apical prolapse repair procedure was performed. Women with infected hematoma were compared to non-infected hematoma with regard to preoperative, intraoperative and postoperative variables with emphasis put on overall prolapse stage, prolapse by compartment and concomitant POP repair and incontinence procedures.

### Statistical analysis

The chi-square and Fischer exact tests were implemented for categorical variables and the *t* test and Mann–Whitney tests for continuous variables. The Mann–Whitney test was implemented for distributions different from normal. All data analysis was performed using statistical software package SPSS 24.0 (SPSS Inc., Chicago, IL). We report odds ratios (OR), 95% confidence interval (CI), and two-sided *p* values, with a value of < 0.05 considered significant.

## Results

During the study period 649 women underwent hysterectomy at our medical center. One case was excluded due to missing data in patient medical records. The abdominal route was chosen for 450 cases (open-70.2%, laparoscopic-29.8% total-79.3%, supracervical-20.7%) as opposed to 198 cases in which vaginal hysterectomy was performed. One-hundred and six women in the abdominal hysterectomy group and 168 women in the vaginal hysterectomy group underwent ultrasound exam following surgery. Pelvic hematoma was diagnosed by imaging in 50 women (7.7%) including 41 women who underwent vaginal hysterectomy and 9 women who underwent abdominal hysterectomy. In 14 (2.2%) cases the hematoma became infected resulting in need for readmission and further treatment.

Data regarding cases of infected pelvic hematoma are presented in Table 1. This group included 9 (64.3%) women who underwent vaginal hysterectomy, all of which were performed for pelvic organ prolapse repair, 4 (28.6%) women who underwent total laparoscopic hysterectomy and one case (7.1%) of total abdominal hysterectomy. The indications for surgery in this group included uterine leiomyoma (3 cases) and malignancy (2 cases).

**Table 1:** Intra-operative and post-operative Infected hematoma data

Type of hysterectomy	
Vaginal hysterectomy	9 (64.3%)
Total abdominal hysterectomy	1 (7.1%)
Total laparoscopic hysterectomy	4 (28.6%)
Antibiotic prophylaxis	
Cefazolin	9 (64.3%)
Cefazolin plus Clindamycin/Gentamycin	2 (14.3%)
Clindamycin	2 (14.3%)
Clindamycin plus Gentamycin	1 (7.1%)
Bacteria identified	8 (57.1%)
Post-operative fever	8 (57.1%)
Days from surgery to fever	9.3 ± 4.2
Post-operative WBC	13.8 ± 3.3
Post-operative platelets	367.5 ± 116.7
Post-operative CRP	8.7 ± 4.0
Hospitalization duration (days)	5.9 ± 3.3
Drainage	9/14 (64.3%)
Cotton swab	7 (77.8%)
Sonographic guided aspiration	2 (22.2%)
Fever duration (days)	1.2 ± 0.4
Days until clinical improvement	2.6 ± 1.5

Data presented as mean ± SD or n(%)

*CRP* C-reactive protein, *WBC* white blood cells

All women with infected hematoma presented with lower abdominal pain. Eight (57.1%) of those women were febrile and 6 (42.8%) had excessive vaginal bleeding which caused them to seek medical attention. Mean time from surgery to diagnosis of infected hematoma was 8.9 days (SD = 4.53). Average hematoma largest diameter of 4.8 (SD = 2.03) centimeters (cm) was measured with most hematomas (n = 12) located above the vaginal cuff. Mean white blood cell count (WBC) on readmission was 13.8 (SD = 3.27) 1000/µL and C-reactive protein (CRP)—8.7 (SD = 4.02) mg/dL. Clinical improvement was noted on average 2.6 (SD = 1.56) days following commencement of antibiotic treatment. For women who presented with fever, mean time to resolution of fever was 1.2 day (SD = 0.41).

In an attempt to assess the effect of hysterectomy route on risk of infected pelvic hematoma, an analysis was performed comparing prevalence of infected hematoma in all vaginal cases compared to the abdominal approach including both open and laparoscopic surgery. Women who underwent vaginal surgery were more likely to return with infected pelvic hematoma compared to women who underwent open abdominal or laparoscopic surgery (4.5% vs. 1.1%, *p* < 0.05).

In order to identify further risk factors for infected pelvic hematoma, a comparison was performed between women diagnosed with an asymptomatic pelvic hematoma and women with an infected pelvic hematoma. Results of this comparison are presented in Tables 2 and 3. Women who underwent vaginal hysterectomy were more likely to develop an infected pelvic hematoma compared to the abdominal and laparoscopic routes (*p* < 0.05). Furthermore, higher preoperative platelet count was associated with an increased risk of infected pelvic hematoma. Other parameters investigated including age, BMI, parity, comorbidities, previous abdominal surgery, prolapse stage, concomitant procedures, surgery length, delta hemoglobin and length of hospital stay were not correlated with increased risk of infected pelvic hematoma.

**Table 2:** Demographic and pre-operative characteristics of the study population—patients with and without infected hematoma

Parameter	Non-infected pelvic hematoma	Infected pelvic hematoma	*p* value
No. of patients	36	14	
Age	61.1 ± 9.3	60.1 ± 11.2	0.756
BMI	27.4 ± 4.1	28.0 ± 4.7	0.665
Menopausal	30 (83.3%)	10 (71.4%)	0.436
Parity	4.9 ± 2.9	3.7 ± 3.2	0.201
No. of vaginal deliveries	4.2 ± 2.8	3.4 ± 3.0	0.419
Smoker	5 (13.9%)	2 (14.3%)	1.00
Comorbidity			
Diabetes	4 (11.1%)	3 (21.4%)	0.384
Hypertension	10 (27.8%)	3 (21.4%)	0.734
Hypothyroidism	5 (13.9%)	6 (42.9%)	0.052
Prior pelvic/abdominal surgery	10 (27.8%)	2 (14.3%)	0.468
Indication for hysterectomy			
Uterine prolapse	32 (88.9%)	9 (64.3%)	0.053
Uterine leiomyoma	1 (2.8%)	3 (21.4%)	
Malignancy	1 (2.8%)	2 (14.3%)	
Menorrhagia	1 (2.8%)	0	
Preventive	1 (2.8%)	0	
Cystocele	32 (88.9%)	9 (64.3%)	0.094
Rectocele	28 (77.8%)	9 (64.3%)	0.474
Urinary incontinence			0.146
No incontinence	19 (52.8%)	10 (71.4%)	
Stress	15 (41.7%)	2 (14.3%)	
Mixed	2 (5.6%)	2 (14.3%)	
Pre-operative hemoglobin (g/dL)	13.2 ± 1.0	13.0 ± 1.1	0.687
Pre-operative platelets (10^9^/L)	241.0 ± 93.4	306.1 ± 72.3	0.035

Data presented as mean + SD or n(%)

*BMI* body-mass index, *POP-Q* pelvic organ prolapse quantification system

**Table 3:** Intra-operative and post-operative characteristics of the study population—patients with and without infected hematoma

Parameter	Non-infected pelvic hematoma	Infected pelvic hematoma	*p* value
No. of patients	36	14	
Type of hysterectomy			0.027
Vaginal hysterectomy	32 (88.9%)	9 (64.3%)	
Total abdominal hysterectomy	2 (5.6%)	1 (7.1%)	
Subtotal abdominal hysterectomy	1 (2.8%)	0	
Total laparoscopic hysterectomy	1 (2.8%)	4 (28.6%)	
Type of anesthesia			0.126
General	34 (94.4%)	11 (78.6%)	
Combined spinal epidural	2 (5.6%)	2 (14.3%)	
Epidural	0	1 (7.1%)	
Concomitant procedures			
Salpingectomy	17 (47.2%)	6 (42.9%)	0.781
Oophorectomy	9 (25.0%)	4 (28.6%)	1.00
Duration of surgery (min)	120.7 ± 35.0	132.7 ± 71.7	0.427
Intraoperative excessive bleeding	1 (2.8%)	0	1.00
Post-operative	4 (11.1%)	2 (14.3%)	1.00
Hematoma location^a^			1.00
Above vaginal cuff	27 (79.4%)	12 (85.7%)	
Anterior to bladder	4 (11.8%)	1 (7.1%)	
Posterior to bladder	3 (8.8%)	1 (7.1%)	
Hospital stay (days)	3.4 ± 2.2	4.1 ± 2.5	0.300
Post-operative hemoglobin (g/dL)	11.1 ± 1.5	11.0 ± 1.2	0.774
Delta hemoglobin (g/dL)	2.1 ± 1.2	2.0 ± 1.3	0.958
Post-operative platelets (10^9^/L)	207.5 ± 61.3	227.6 ± 52.8	0.285
Post-operative fever	1 (2.8%)	7 (50.0%)	< 0.001
Mean hematoma size (mm^2^)	1390.7 ± 1698.1	1905.6 ± 1806.8	0.366
Hematoma largest diameter (mm)	39.1 ± 18.2	48.1 ± 20.3	0.140

Data presented as mean + SD or n(%)

*WBC* white blood cells

^a^Data was available for 34/36 cases

Further subgroup analysis was performed with regard to women who underwent vaginal hysterectomy for treatment of POP. This group included 41 women who underwent vaginal hysterectomy with uterosacral ligament suspension. Women with asymptomatic pelvic hematoma were compared to those with infected pelvic hematoma (Table 4). Lower parity was associated with higher risk of infected pelvic hematoma (*p* < 0.05). Other variables investigated did not reach statistical significance including preoperative prolapse stage, concomitant surgeries, length of surgery and hematoma location. A trend was noted between larger hematoma size and risk of infected pelvic hematoma (*p* = 0.086). Preoperative platelet count was higher in the infected hematoma group but failed to reach statistical significance (*p* = 0.083).

**Table 4:** Demographic, pre-operative, intra-operative and post-operative characteristics of the study population—vaginal hysterectomy with pelvic prolapse repair only

Parameter	Non-infected pelvic hematoma	Infected pelvic hematoma	*p* value
No. of patients	32	9	
Age	61.9 ± 8.9	63.4 ± 11.2	0.675
BMI	27.1 ± 4.1	27.8 ± 5.8	0.684
Menopausal	28 (87.5%)	8 (88.9%)	0.910
Parity	5.0 ± 2.8	3.0 ± 1.3	0.027^a^
Smoker	3 (9.4%)	0	1.00
Comorbidity	16 (50.0%)	3 (33.3%)	0.376
Prior pelvic/abdominal surgery	10 (31.3%)	2 (22.2%)	0.702
Cystocele	32 (100.0%)	9 (100.0%)	1.00
Rectocele	28 (87.5%)	9 (100.0%)	0.559
Urinary incontinence			0.198
No incontinence	15 (46.9%)	5 (55.6%))	
Stress	15 (46.9%)	2 (22.2%)	
Mixed	2 (6.3%)	2 (22.2%)	
Pre-operative POP-Q stage^b^			0.999
1	0	0	
2	1 (3.1%)	0	
3	26 (83.9%)	8 (88.9%)	
4	4 (12.5%)	1 (11.1%)	
Ba	4.0 ± 2.9	4.9 ± 2.4	0.421
C	2.3 ± 4.2	3.8 ± 3.4	0.370
Bp	0.9 ± 3.6	-0.5 ± 3.0	0.347
Type of anesthesia			0.061
General	30 (93.8%)	6 (66.7%)	
Combined spinal epidural	2 (6.3%)	2 (22.2%)	
Epidural	0	1 (11.1%)	
Concomitant procedures			
TVT/TVTO	21 (65.6%)	6 (66.7%)	1.00
Anterior repair	32 (100.0%)	8 (88.9%)	0.220
Posterior repair	29 (90.6%)	8 (88.9%)	1.00
Perineorrhaphy	8 (25.0%)	2 (22.2%)	1.00
Salpingectomy	15 (46.9%)	1 (11.1%)	0.066
Oophorectomy	7 (21.9%)	0	0.315
Duration of surgery (min)	125.6 ± 29.3	122.7 ± 28.3	0.789
Intraoperative excessive bleeding	1 (3.1%)	0	1.00
Post-operative data	3 (9.4%)	2 (22.2%)	0.299
Hematoma location^b^			1.00
Above vaginal cuff	25(83.3%)	8 (88.9%)	
Anterior to bladder	2 (6.7%)	1 (11.1%)	
Posterior to bladder	3(10.0%)	0 (0%)	
Hospital stay (days)	3.2 ± 2.2	3.7 ± 2.1	0.565
Post-operative hemoglobin (g/dL)	11.1 ± 1.4	10.9 ± 1.1	0.648
Delta hemoglobin (g/dL)	2.0 ± 1.0	2.3 ± 1.4	0.486
Post-operation platelets (10^9^/L)	206.6 ± 60.2	223.9 ± 62.6	0.454
Mean hematoma size (mm^2^)	1179.0 ± 778.3	2148.1 ± 2173.9	0.252
Hematoma largest diameter (mm)	37.2 ± 12.3	51.9 ± 22.4	0.086^a^

Data presented as mean ± SD or n(%)

^a^Mann–Whitney Test significance

^b^Data was available for majority of cases

*POP-Q* pelvic organ prolapse quantification system, *Ba* point B anterior, *Bp* point B posterior, *C* cervix or vaginal cuff, *TVT* tension-free vaginal tape, *TVT-O* tension-free obturator tape

Data regarding preoperative antibiotic prophylaxis were available for all cases. Different antibiotic prophylactic regimens were administered including cefazolin (n = 37), clindamycin (n = 7), clindamycin and gentamycin (n = 3), cefazolin and gentamycin (n = 1) and clindamycin and ciprofloxacin (n = 1). Further subgroup analysis was performed comparing regimens including cefazolin as opposed to clindamycin. No difference with regard to risk of infected pelvic hematoma was noted between the groups.

**Fig. 1:**
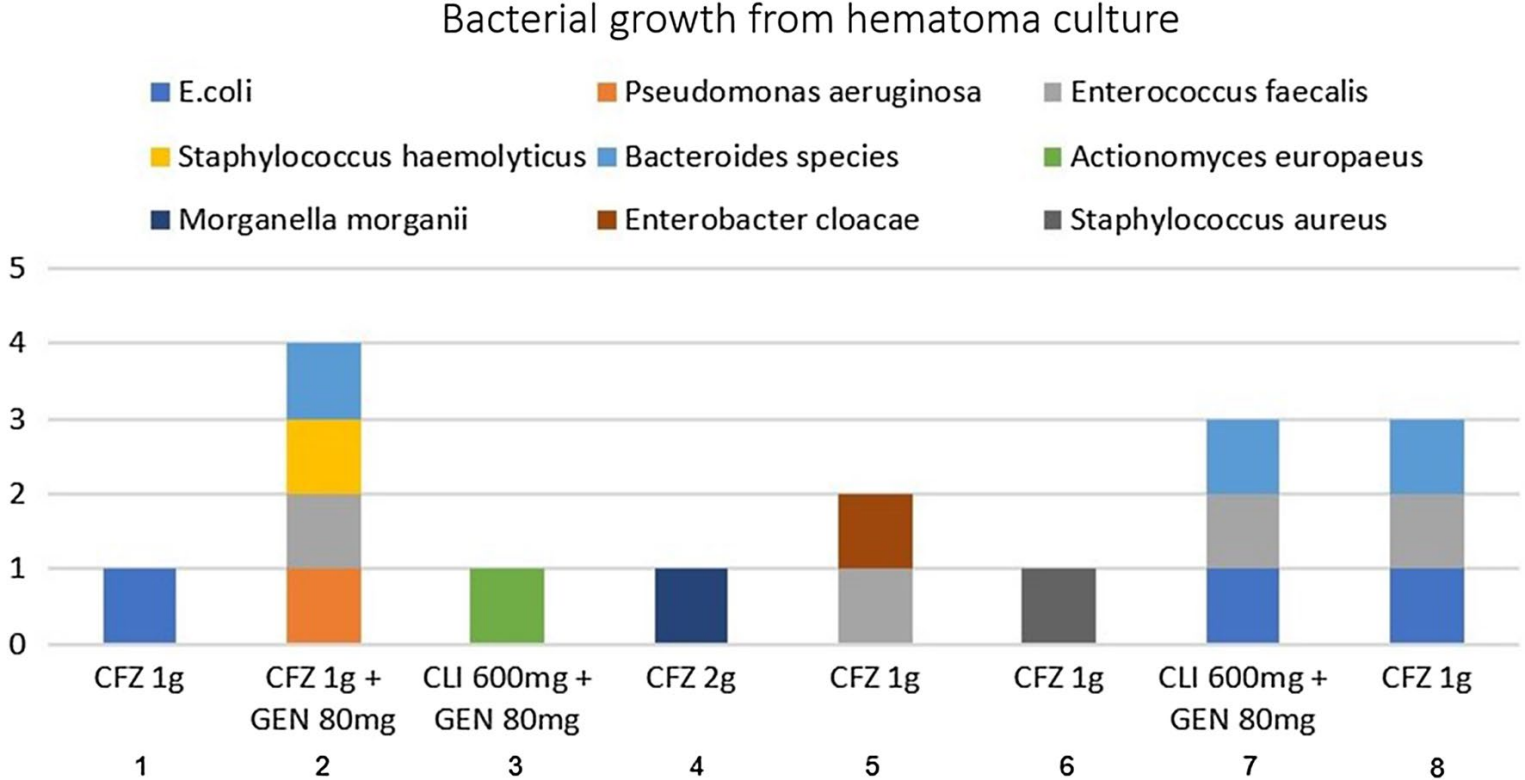
Bacterial growth from hematoma culture. Bacterial growth from hematoma culture in 8 women diagnosed with infected pelvic hematoma. Antibiotic prophylactic regimen administered to each patient prior to surgery is depicted as well. *CFZ* cefazolin, *GEN* gentamycin, *CLI* clindamycin

In 8 cases bacterial culture was positive from the hematoma which was drained either spontaneously or following surgical procedure. Information regarding these cases is presented in Fig. 1. For most cases drainage was performed by introducing a sterile cotton swab through the sutures of the vaginal cuff but for two cases drainage under ultrasound guidance was required. *Enterococcus faecalis* was the most common bacteria to grow in culture (n = 4) while in four of the cases there was more than one bacterial species.

## Discussion

Pelvic hematoma following hysterectomy is a common finding which often goes undiagnosed. In contrast, infected pelvic hematoma is rare but when present causes great discomfort for women and poses a clinical challenge for caregivers. We have shown prevalence of pelvic hematoma and infected hematoma to be 7.7% and 2.2% respectively. Vaginal route of hysterectomy was shown to be associated with higher risk for infected pelvic hematoma as compared to the abdominal route (4.5% and 1.1% respectively *p* < 0.05). A higher preoperative platelet count was also associated with increased risk of infected hematoma, but the clinical importance of this finding remains unclear. Other factors assessed did not reach statistical significance.

Previous studies have been equivocal regarding the prevalence of pelvic hematoma ranging between 25% and 98% [2, 3]. This is probably due to under-diagnosis of asymptomatic hematomas. In our study all women who underwent vaginal hysterectomy, all treated for POP, had an ultrasound exam performed following surgery for post-void residual measurement. This enabled us to identify women with pelvic hematoma, which would have gone otherwise undetected.

To our knowledge, this is the first study to compare women with infected hematoma to women with asymptomatic pelvic hematoma. Similar to the findings of Segev et al. [4] vaginal route was associated with increased risk of infected pelvic hematoma. We also compared infected and non-infected hematoma in the vaginal hysterectomy group alone. In this group, lower parity was associated with higher risk of infection but this cannot be explained by pelvic organ prolapse stage, which was not significantly different between the groups. A trend towards association between larger hematoma size and infection was also evident. Previous studies have suggested that hematoma size may be a risk factor for infection but this variable has never been evaluated in a cohort directly comparing infected to non-infected hematomas.

On performance of vaginal hysterectomy with apical suspension there seems to be increased risk of bleeding compared to hysterectomy without POP repair. In a large retrospective study, Heisler et al. [9] found that the addition of pelvic reconstruction to vaginal hysterectomy was associated with increased morbidity as compared to vaginal hysterectomy alone. Furthermore, native tissue repair using the vaginal fibro-muscular layer requires development of a non-anatomical plain containing abundant vasculature which may lead to excessive bleeding. This may account for the higher rate of infected hematoma in this group though the specific role that apical suspension plays in this is still unclear.

During total hysterectomy, the peritoneum is opened communicating the vagina and the abdominal cavity. This newly formed connection though temporary, affords an opportunity for polymicrobial flora of aerobes and anaerobes normally colonizing the vagina to enter the abdominal cavity and cause infection. Vaginal hysterectomy procedure involves peritoneal entry at an early stage, thus the duration of exposure of the peritoneal cavity to vaginal flora is extended. Furthermore, when performing an apical suspension procedure, the exposure time is prolonged. In addition, the surgeon must use surgical instruments, extending from the vagina into the abdominal cavity in order to secure sutures to the relevant pelvic structure. These manipulations may increase the risk of superimposed infection should a pelvic hematoma form.

Most cases of infection following hysterectomy include surgical site involvement which in turn may implicate either skin or the vaginal cuff. Though both are a consequence of bacterial infection, the specific pathogens differ between the two sites. While skin infections are mostly caused by skin flora such as streptococci species, *Staphylococcus aureus* and coagulase negative staphylococci, vaginal infections are strongly affected by the polymicrobial environment present in the vagina.

Avoiding surgical site infection is based on sterile measures, proper cleansing of the surgical site and antibiotic prophylaxis. Cleansing of the vagina prior to hysterectomy includes use of either chlorhexidine solution or iodine based solutions with or without a detergent. A large Swedish cohort reported on higher postoperative infection rates for women who underwent vaginal cleansing with saline compared to chlorhexidine prior to vaginal hysterectomy [10]. Another study compared povidone iodine to chlorhexidine prior to vaginal hysterectomy and found chlorhexidine reduces bacterial load to some extent but these regimens were not evaluated with regard to clinical infection rate [11]. In our department common practice includes vaginal cleansing with iodine based solution for vaginal hysterectomy. Though both aerobic and anaerobic bacteria are considered susceptible to iodine based solutions, the anatomical structure and polymicrobial environment of the vagina limit the ability to sterilized the surgical field.

Current recommendation for preoperative antibiotic prophylaxis before hysterectomy not withstanding surgical route is single dose of a first generation cephalosporin [8]. These recommendations are based on over 30 prospective randomized clinical trials as well as a Cochrane review which showed reduction in postoperative infections including pelvic infection in women who received antibiotic prophylaxis compared to placebo [12]. Furthermore, Data from the Michigan Surgical Quality Collaborative found that women who received beta-lactam alternatives had increased risk of any surgical site infection (both abdominal and vaginal) compared to women who were treated with beta-lactam prophylaxis prior to hysterectomy (OR 1.62, *p* < 0.001) [13]. Beta-lactam alternatives included combinations such as clindamycin plus gentamycin and metronidazole plus gentamycin. In our study no difference was found between antibiotic prophylactic regimens used with regard to risk of infected hematoma but the study group was underpowered to detect such differences.

In our study, we report on 8 women who had a bacterial growth from hematoma culture. As expected, bacteria included normal genital habitants including enterobacteriales, anaerobes and enterococci, some of which are considered inherently resistant to the common prophylactic regimen, id est first generation cephalosporins. *Enterococcus faecalis*, the most abundant pathogen isolated in our patients is not only resistant to the common prophylaxis, but also to most empiric treatment protocols [14]. These findings raise the possibility of alternative antibiotic regimens which may give better coverage for possible anaerobic pathogens. As mentioned earlier previous studies have pointed towards the superiority of beta-lactam prophylaxis with regard to risk of infection following hysterectomy [12, 13]. These studies did not differentiate between skin infections and vaginal cuff infections. Additionally, no regimens including both beta-lactam antibiotics and anaerobic coverage, were tested. Some reports have suggested amoxicillin-calvulanic acid may have increased efficacy in treatment of gynecologic infections following hysterectomy compared to first generation cephalosporins but this has yet to be validated with regard to prophylactic treatment [15].

Apart from its retrospective nature the study has certain limitations. The ability to detect possible risk factors was limited due to the small study group. Data regarding hematoma measurement were missing a third dimension rendering calculation of volume difficult. Due to the routine practice of ultrasound exam following pelvic organ prolapse repair more asymptomatic hematomas were diagnosed in the vaginal hysterectomy group. In contrast, some women in the abdominal hysterectomy group with asymptomatic hematomas probably went undetected and were lost to follow up. While this enabled us to detect hematomas which in previous studies would have gone unnoticed, we could not draw conclusions regarding prevalence of asymptomatic hematomas in abdominal hysterectomy.

The strengths of the study include presentation of a large cohort with regard to this infrequent complication. In addition, we had a unique opportunity to look at asymptomatic hematomas and determine the risk of infection, at least in the vaginal hysterectomy group. To our knowledge, this is the first study to report on growth of specific organisms from bacterial culture originating from the hematoma site. Preoperative and postoperative data included use of standardized measures such as the POP-Q system and laboratory findings.

## Conclusion

Infected hematoma following hysterectomy is an uncommon complication with increased risk of occurrence following vaginal compared to abdominal hysterectomy. We also found most of these infections to be caused by anaerobic bacteria. We believe these findings may help clinicians to better counsel women regarding the risks of surgery and may pave the way for future studies in pursuit of the optimal antibiotic prophylactic regimen prior to hysterectomy.

## Data Availability

The datasets used and/or analyzed during the current study are available from the corresponding author on reasonable request.

## Abbreviations

POP: Pelvic organ prolapse
POP-Q: Pelvic organ prolapse quantification system
WBC: White blood cell count
